# Comparative analysis of the testes from wild-type and *Alkbh5*-knockout mice using single-cell RNA sequencing

**DOI:** 10.1093/g3journal/jkac130

**Published:** 2022-06-02

**Authors:** Shihao Hong, Xiaozhong Shen, Chunhai Luo, Fei Sun

**Affiliations:** Institute of Reproductive Medicine, Medical School of Nantong University, Nantong 226001, China; Institute of Reproductive Medicine, Medical School of Nantong University, Nantong 226001, China; Institute of Reproductive Medicine, Medical School of Nantong University, Nantong 226001, China; Institute of Reproductive Medicine, Medical School of Nantong University, Nantong 226001, China

**Keywords:** single-cell RNA-seq, spermatogenesis, *Alkbh5*, mouse testis, m6A methylation

## Abstract

The RNA demethylase ALKBH5 is regarded as the “eraser” in N6-methyladenosine modification. ALKBH5 deficiency causes male infertility in mice; however, the mechanisms that confer disruption of spermatogenesis are not completely clear. In this study, we profiled testis samples from wild-type and *Alkbh5*-knockout mice using single-cell RNA sequencing. We obtained single-cell RNA sequencing data of 5,596 and 6,816 testis cells from a wild-type and a knockout mouse, respectively. There were differences detected between the transcriptional profiles of the groups at various germ cell developmental stages. This ranged from the development of spermatogonia to sperm cells, in macrophages, Sertoli cells, and Leydig cells. We identified the differentially expressed genes related to spermatogenesis in germ cells and somatic cells (Sertoli cells and Leydig cells) and evaluated their functions and associated pathways, such as chromatin-related functional pathways, through gene ontology enrichment analysis. This study provides the first single-cell RNA sequencing profile of the testes of ALKBH5-deficient mice. This highlights that ALKBH5 is an important gene for germ cell development and spermatogenesis and offers new molecular mechanistic insights. These findings could provide the basis for further research into the causes and treatment of male infertility.

## Introduction

Mammalian spermatogenesis is a complex process of cell differentiation. The process is divided into 3 main stages: mitosis of spermatogonia, meiotic division of spermatocytes, and spermiogenesis (de Rooij 2001; Griswold 2016). In the mitosis stage, spermatogonia produce 2 types of cells through mitosis. One type of cell does not enter the spermatogenesis cycle and retains the ability of mitosis, whereas the other type differentiates and gives rise to primary spermatocytes (Lin and Tong 2019). Next, primary spermatocytes become secondary spermatocytes through meiosis I, and the secondary spermatocytes then form round spermatids through meiosis II (Handel and Schimenti 2010). Finally, the round spermatids differentiate into elongated spermatids accompanied by extensive morphological changes, transforming into mature sperms (Kumar *et al.* 2018).

Gene expression during spermatogenesis is finely regulated at both the transcriptional and posttranscriptional levels. At both of these levels, RNA modification is an extremely important regulatory process. N6-methyladenosine (m6A) is the most common nucleotide modification in mRNA (Yue *et al.* 2015) and is critical during spermatogenesis. Knockout (KO) mice lacking the m6A writer (*Metll3*) (Xu *et al.* 2017; Yang *et al.* 2017), eraser (*Alkbh5*) (Zheng *et al.* 2013; Tang *et al.* 2018), or reader (*Ythdc2*) (Hsu *et al.* 2017; Wojtas *et al.* 2017) genes have dysfunctions in spermatogenesis that lead to male infertility. Among these genes, the mammalian RNA demethylase *Alkbh5* shows the highest expression in the mouse testes and is related to RNA metabolism and mouse fertility. Global inactivation of the *Alkbh5* gene results in males that are infertile due to apoptosis in the spermatogenic tubules (Zheng *et al.* 2013). Male mice deficient in ALKBH5 showed increased m6A markers in the RNA (Zheng *et al.* 2013). KO mice appeared anatomically normal and lived into adulthood but exhibited impaired spermatogenesis with compromised production of pachytene spermatocytes and round spermatids (Zheng *et al.* 2013). The m6A demethylation dependent on *Alkbh5* controls the splicing and stability of long 3′-untranslated region mRNAs in male germ cells. KO mice have smaller testes, the morphology of seminiferous tubules is disordered, and the number of sperm cells decreases significantly (Tang *et al.* 2018). However, the mechanism by which *Alkbh5* exerts these effects remains unclear.

The transcriptome of *Alkbh5* KO and wild-type (WT) mice has not yet been compared because of the challenges in analyzing bulk RNA-seq data with such samples. Single-cell RNA sequencing (scRNA-seq) technology has an advantage over bulk sequencing with whole-tissue analysis. It enables the evaluation of gene expression at the single-cell level and offers unprecedented insight into cellular heterogeneity (Suzuki *et al.* 2019). The morphological changes in *Alkbh5* KO mice have been identified; however, the mechanisms at the single-cell level that leads to spermatogenesis disorders have not been elucidated. To better understand this mechanism, in this study, we performed the scRNA-seq analysis of 2 testes: one from a WT adult mouse with normal fertility and another from a mouse with *Alkbh5* KO and exhibiting fertility defects. We compared the gene expression levels and associated functions of the genes differentially expressed between the 2 groups at the single-cell level, to understand the influence of ALKBH5 protein deficiency on mouse spermatogenesis.

## Materials and methods

### Animals and treatment

All animal experiments in this study were approved by the Institutional Review Board of Nantong University (Nantong, China). Mice were housed under specific pathogen-free conditions in a temperature- and humidity-controlled animal facility, with a 12-h:12-h light:dark cycle (6:00 AM to 6:00 PM). *Alkbh5*^+/−^ mice were a gift from Dr Yamei Niu, Institute of Basic Medical Sciences, Chinese Academy of Medical Science, and were purchased from Jackson Labs. Heterozygous *Alkbh5* mice were crossed to produce homozygous *Alkbh5* mice. WT and KO mice were sacrificed at 12 weeks and the testes were harvested to be measured, weighed, and photographed.

### Western blot assay

The testes from 12-week-old adult WT and KO mice were lysed using RIPA lysis buffer. The protein concentration was quantified using the BCA Protein Assay Kit (P10012; Beyotime Biotechnology). Equal amounts of the total proteins were separated on a 12% SDS-PAGE and then transferred onto a polyvinylidene difluoride membrane. The membranes were blocked in 5% skim milk solution for 1 h at 37°C and then incubated overnight with primary anti-Alkbh5 (1:500, HPA007196; Sigma) and β-tubulin (1:3,000, ab108342; Abcam) at 4°C. The following day, the membranes were washed with PBS 3 times and incubated with goat anti-rabbit HRP (1:5,000, ab205718; Abcam) at 20°C for 1 h. Proteins were finally visualized using an Odyssey infrared imaging system (Li-Cor Biosciences).

### Histological analysis

Testes and epididymis of WT and KO mice were fixed in 4% paraformaldehyde overnight at 4°C and then embedded in paraffin. The tissues were cut into 5-μm-thick sections, mounted on glass slides, and deparaffinized. The testicular sections were stained with periodic acid–Schiff (PAS) and Epididymal sections were stained with hematoxylin and eosin (H&E). Standard light micrographs were taken using a microscope (DM2000; Leica).

### Immunofluorescence

Paraffin sections of the testes were boiled in sodium citrate buffer (0.01 M, pH 6.0) for 10 min. The sections were blocked with 5% BSA in PBST for 1 h at 37°C and then incubated with primary anti-Alkbh5 (1:150, HPA007196; Sigma) antibodies at 4°C overnight. The sections were washed with PBS and then incubated with goat anti-rabbit Alexa Fluor 488 (1:500, A11034; Thermo Fisher) at 37°C for 1 h. Finally, slides were mounted using DAPI and viewed under the fluorescent microscope (Axio Imager M2; Zeiss).

### TUNEL assay

Testicular sections (20 µm) from 12-week-old adult WT and KO mice were used for TUNEL staining, according to the manufacturer’s instructions (TUNEL BrightRed Apoptosis Detection Kit, A113-0; Vazyme). The testicular sections were incubated with proteinase K for 20 min at 37°C and then with equilibration buffer (1× TdT) for 30 min at 37°C. Finally, TUNEL reaction mixture and DAPI were used for staining, and the images were visualized with a fluorescence microscope (Axio Imager M2; Zeiss). Random seminiferous tubules (*n* = 100) were quantified in the TUNEL-positive cells from each testis.

### Real-time quantitative PCR

Total RNA from WT and KO mice testicular tissues were extracted by TRIzol reagent (15596026; Thermo Fisher). Then, 1 μg of total RNA was reverse transcribed using the cDNA Reverse Transcription kit (6110A; Takara). Quantitative Real-Time PCR was performed using TB Green™ Premix Ex Taq™ II (RR420A; Takara) on a LightCycler 96 Real-Time PCR System (Roche), according to the manufacturer’s instructions. All primers for genes are listed in Supplementary Table 1 and were synthesized by Genewiz (Suzhou, China). Relative abundance of mRNA expression was calculated with the 2^−ΔΔCt^ method and normalized to β-actin mRNA expression levels.

### Tissue dissociation and preparation

Three unilaterally decapsulated testes were dissected from 3 adult WT and 3 adult KO mice. The fresh testicular tissues were stored in the sCelLiveTM Tissue Preservation Solution (Singleron Biotechnologies) on ice within 30 min after the surgery. The tissues were washed with Hanks’ Balanced Salt Solution thrice and then digested with 2 ml sCelLiveTM Tissue Dissociation Solution (Singleron Biotechnologies) using the Singleron PythoN™ Automated Tissue Dissociation System (Singleron Biotechnologies) at 37°C for 15 min. The tissue lysate was centrifuged at 500 × *g* for 5 min, and the pellet was suspended gently with PBS. The samples were stained with trypan blue (T8154; Sigma), and the cellular viability was evaluated microscopically. Apoptotic or dead cells were discarded.

### Library construction and scRNA-seq

Single-cell suspensions were prepared at 1 × 10^5^ cells/ml in phosphate-buffered saline and loaded onto microfluidic devices (microfluidic chip; 1CellBio). The sequencing libraries were constructed with GEXSCOPE Single-Cell RNA Library Kit (Singleron Biotechnologies), according to the manufacturer’s instructions. The Qubit 2.0 fluorometer was used for preliminary quantification. The library was diluted to 1.5 ng/μl. The Agilent 2100/4200 bioanalyzer was used to detect the insert size of the library. After confirming that the insert size met the expectation, the effective concentration of the library was measured using quantitative polymerase chain reaction. Accurate quantification (the effective concentration of the library was higher than 2 nM) ensured the quality of the library. The different libraries were pooled, according to the effective concentration and quantity of target data for Illumina sequencing. The sequencing-by-synthesis principle was then applied. Fluorescently labeled dNTP and DNA polymerase were added to the flow cell for sequencing, and adaptor primers were used for amplification. After each sequencing, cluster extended the complementary strand, each time a fluorescently labeled dNTP was added to release the corresponding fluorescence, the sequencer captured the fluorescent signal and converted the light signal into a sequencing peak through computer software to obtain the sequence information of the fragment to be tested. Individual libraries were diluted to 4 nM and pooled for sequencing. The pools were sequenced on Illumina HiSeq X with 150-bp paired end reads at an average sequencing depth of 50K reads per sample.

### Raw data processing

The raw sequencing data were processed using an internal pipeline. Briefly, after filtering read 1 without poly T tails, cell barcode and UMI were extracted. Adapters and poly A tails were trimmed (fastp V1) before aligning read 2 to GRCh38 with ensemble version 92 gene annotation (fastp 2.5.3a and featureCounts 1.6.2) (Liao *et al.* 2014). Reads with the same cell barcode, UMI, and gene were grouped together to calculate the number of UMIs per gene per cell. The UMI count tables of each cellular barcode were used for further analysis. Cell-type identification and clustering analysis were performed using Seurat program (Butler *et al.* 2018).

### Downstream data analysis

The scRNA-seq data were analyzed using the pipeline of the R package Seurat (https://satijalab.org/seurat/, v.3.2.2) for the cell identification and clustering analysis (Butler *et al.* 2018; Stuart *et al.* 2019). The UMI counts matrix was loaded into R session using the function Read10X. There were 2 Seurat objects that were built respectively from the KO and WT samples. Cells that expressed more than 500 genes were retained. The standard workflow of Seurat V3 was employed to alleviate the batch effects and integrate data (Chothani *et al.* 2019). A total of 2,000 top variable features were identified after normalizing the UMI counts matrix. The Seurat function FindIntegrationAnchors was used and 20 dimensions were selected as parameters. The anchors were identified and data were integrated using the function IntegrateData. The subsequent steps followed the standard workflow for visualization and clustering applied to single samples. The function ScaleData was used for scalation of the matrix and 30 principal component dimensions were chosen for uniform manifold approximation (UMAP) and FindNeighbors analysis. After these steps, the function FindClusters was used to perform clustering analysis in the samples, with the parameter resolution set to 0.3. Previously established markers were used for the identification of each cell type (Jung *et al.* 2019). The function FindMarkers was used to identify the genes differentially expressed in the WT and KO samples, with the parameter min.pct set to 0.25. Gene ontology (GO) enrichment analysis of the differentially expressed genes was performed using the R package clusterProfiler (v 3.12.0) (Yu *et al.* 2012). Germ cells from KO samples were regarded as input and was preprocessed using Dynamo (version 1.0.0), and then, the velocity, differentiation potential, and acceleration of cells were analyzed (Qiu *et al.* 2022). Cell contact was analyzed by CellChat (version 1.1.3), and the raw expression matrices of KO samples and WT samples were regarded as input (Jin *et al.* 2021).

## Results

### *Alkbh5* KO causes impaired spermiogenesis in mice

**Fig. 1.**
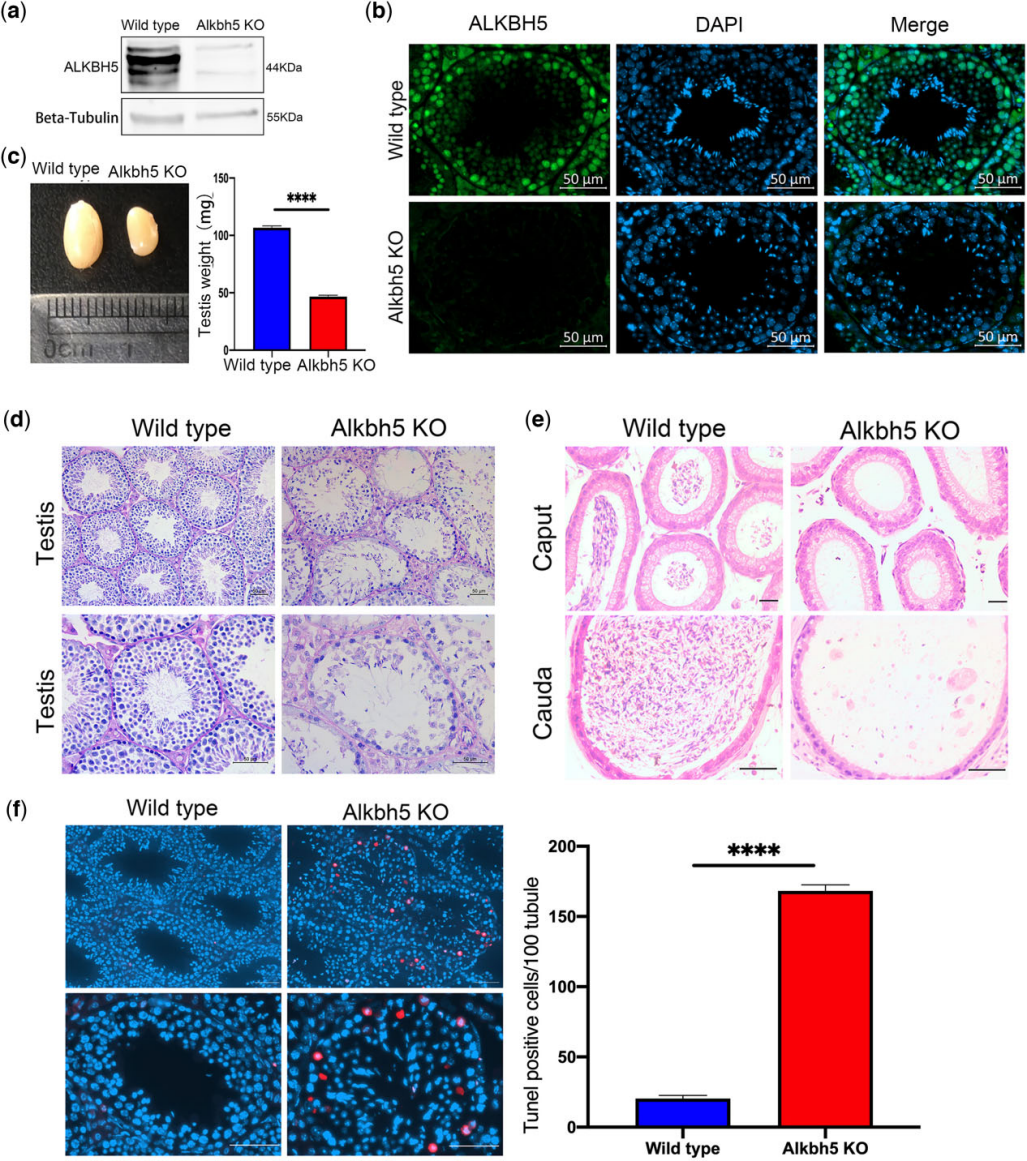
a) Western blots showing the efficiency of ALKBH5 protein KO. β-Actin served as a loading control. b) Immunofluorescence images of testis from 12-week-old WT and *Alkbh5* KO mice stained for ALKBH5 (green) and DAPI (blue) (scale bars: 50 μm). c) Left panel: representative testes gross morphology of 12-week-old WT control and age-matched *Alkbh5 KO* mice. Right panel: testis weights from WT and *Alkbh5* KO mice (*n* = 8 per group; ****P < 0.0001, Student’s *t*-test, error bars are ± SEM). d) Histological analysis with PAS of WT and *Alkbh5* KO mice testis (scale bars: 50 μm). e) WT and *Alkbh5* KO mice caput and cauda epididymis sections stained with H&E (scale bars: 50 μm). f) Left panel: immunofluorescence staining of TUNEL in WT and *Alkbh5* KO mice testis stained for TUNEL (red) and DAPI (blue) (scale bars: 50 μm). Right panel: quantification of TUNEL-positive cells in 100 seminiferous tubules from WT and *Alkbh5* KO mice (*n* = 3 per group; ****P < 0.0001, Student’s *t*-test, error bars are ± SEM).

*Alkbh5* mRNA is the highest expressed in the mice testes, and the global inactivation of the *Alkbh5* gene results in infertile males (Zheng *et al.* 2013). To examine whether *Alkbh5* gene was successfully knocked out, we performed western blot analysis to validate the absence of ALKBH5 protein in KO mouse testes (Fig. 1a). Target gene KO was additionally confirmed by immunofluorescence staining (Fig. 1b). Adult male *Alkbh5* homozygous KO mice were infertile. The testes of KO mice were smaller in shape and of lower quality compared to those of the WT mice (Fig. 1c). PAS staining of testis sections for histological analysis showed that testes of KO mice had increased vacuolation and a reduced number of spermatogenic cells in the seminiferous tubules (Fig. 1d). Hematoxylin and eosin staining of the caput and cauda epididymis revealed that the seminiferous tubules in KO mice had lost a substantial number of germ cells and contained few or no mature spermatozoa in the lumen (Fig. 1e). TUNEL staining further confirmed the significant increase in the numbers of apoptotic cells in the KO mouse testis (Fig. 1f). This phenotypic analysis revealed that ALKBH5 protein is essential for normal spermatogenesis in mice, in line with previous findings.

### Single-cell transcriptional profiling of WT and KO testes and cell-type identification

**Fig. 2.**
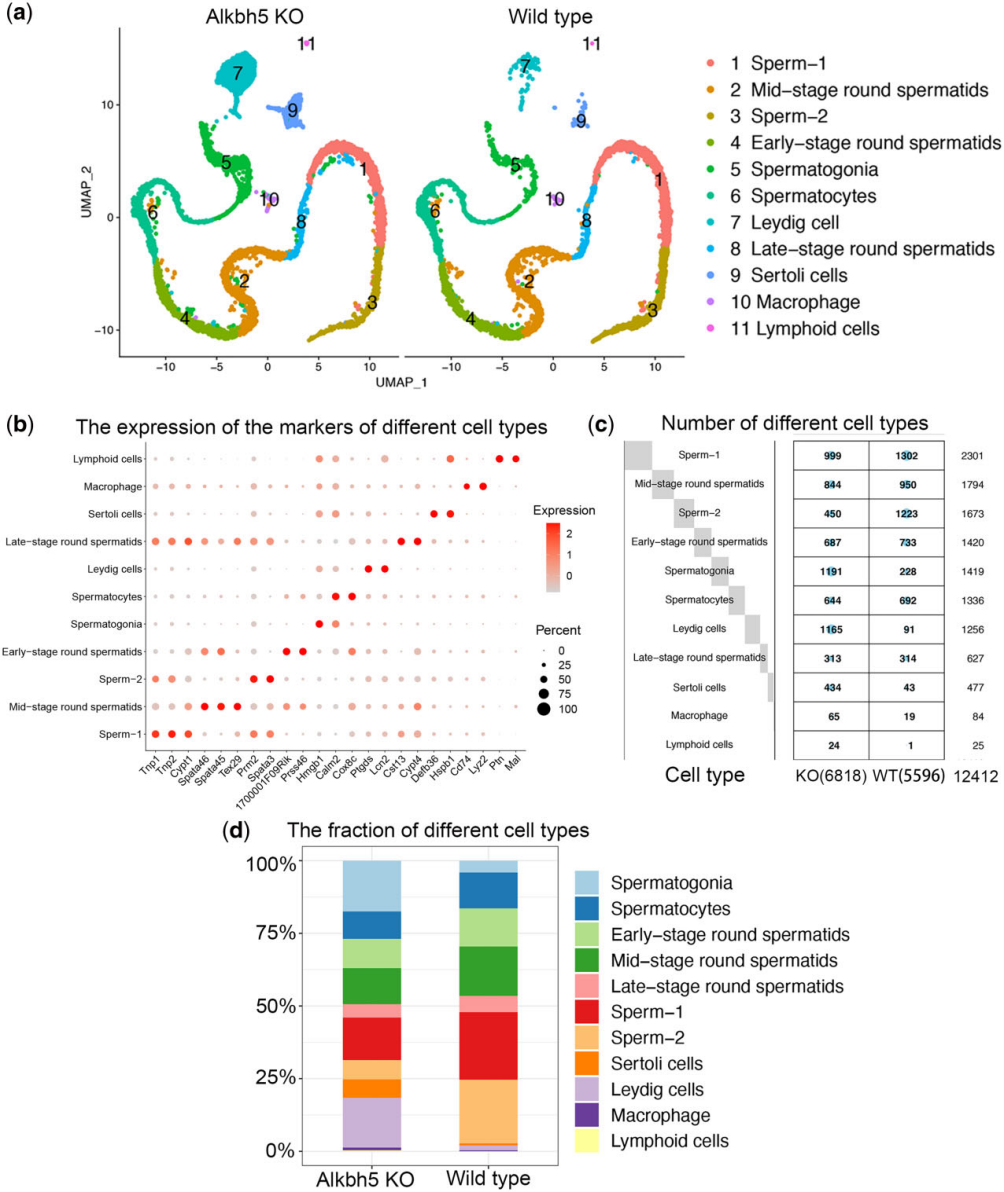
a) Visualization of scRNA-seq data by UMAP plots from WT (*n* = 5,596) and *Alkbh5* KO (*n* = 6,816) mice testis. UMAP projection of *Alkbh5* KO mice testis sample (left) and WT mice testis sample (right). b) Dot plot showing the expression of well-known gene markers in different cell types. c) Plot showing the calculation of the number of different cell types. The larger the blue dot, the greater the number of genes. d) Bar chart summarizing the fraction of different cell types in WT and *Alkbh5* KO mice testis.

After quality control, we obtained 5,596 cells from the WT testis sample and 6,816 cells from the KO testis sample (Supplementary Fig. 1). Canonical correlation analysis, dimensionality reduction, and UMAP identified 11 main cell types based on the reported marker genes for the various cell types in the testis (Jung *et al.* 2019), including the germ cell types, from the spermatogonia to sperm, and the somatic cell types, including Leydig and Sertoli cells (Fig. 2, a and b, Supplementary Fig. 2, and Supplementary Table 2). The proportion and number of Leydig cells and Sertoli cells in the KO sample far exceeded those in the WT sample (Fig. 2c). In addition, the number and proportion of immune cells differed between the groups, with an increase in these cells in the KO sample (Fig. 2d).

### Differential gene analysis of germ cells revealed the mechanism of impaired spermatogenesis in the KO testis

**Fig. 3.**
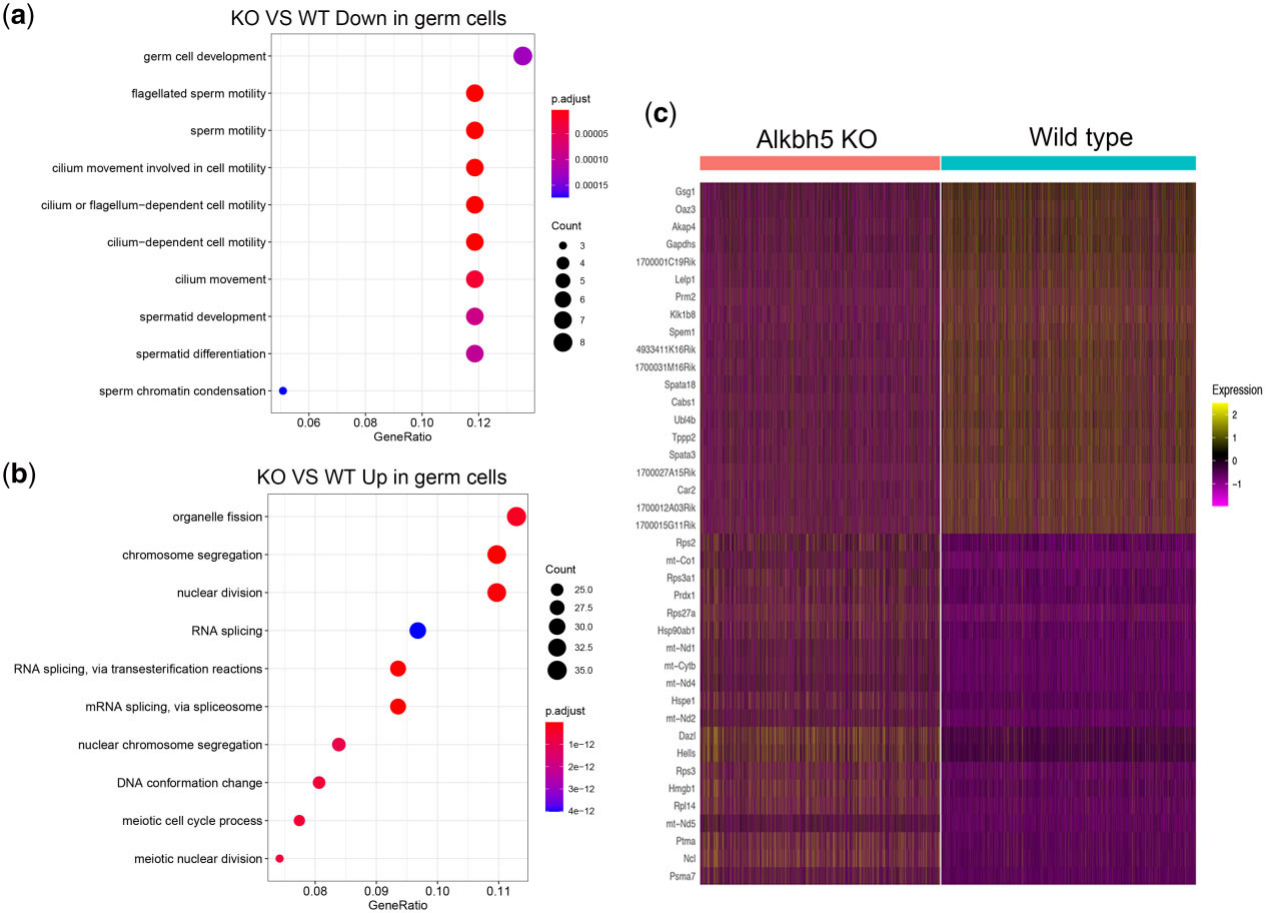
a) Biological process enrichment analysis of genes with downregulated germ cells in *Alkbh5* KO mice compared with WT mice. b) Biological process enrichment analysis of genes with upregulated germ cells in *Alkbh5* KO mice compared with WT mice. c) Heatmap of the top 20 differentially expressed genes in *Alkbh5* KO mice compared with WT mice.

To further analyze how knocking out *Alkbh5* affects the spermatogenesis process in the germ cells, we extracted germ cells from the datasets for differential gene expression analysis on the KO and WT samples. The KO mice had 339 significantly upregulated genes and 80 downregulated genes, compared to that in the WT mice. Gene enrichment analysis of the differentially expressed genes showed that the downregulated genes in the KO sample reflected a slower developmental ability of germ cells compared to that in the WT sample (Fig. 3a and Supplementary Fig. 3). Moreover, the upregulated genes in the KO sample were mainly enriched in mRNA and chromatin-related functional pathways (Fig. 3b and Supplementary Fig. 4). A large proportion of the upregulated genes in the KO samples were mitochondrial and ribosome-related genes (Fig. 3c). These findings suggested that ALKBH5-mediated m6A demethylation could play an important role in regulating chromatin opening, and its abnormal regulation leads to male infertility.

### Dynamo analysis revealed the abnormality of germ cell differentiation

**Fig. 4.**
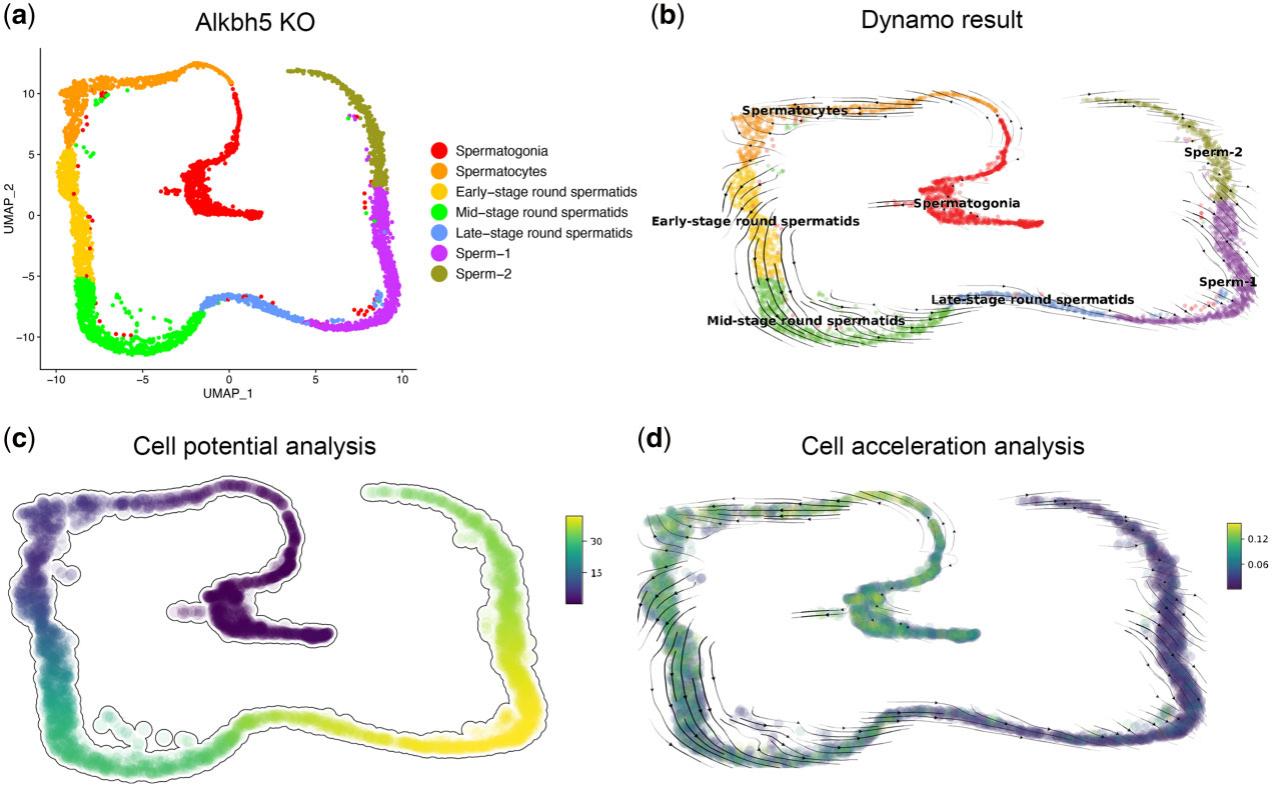
a) UMAP visualization of germ cells in KO mice. b) Visualization of RNA velocity of germ cell differentiation. c) Visualization of cell potential analysis of germ cells. d) Visualization of cell acceleration of germ cell differentiation.

To further explore the differentiation process of germ cells after *Alkbh5* KO. We first extracted germ cells from KO dataset and then used Dynamo to simulate and analyze the differentiation process of cells after dimension reduction (Fig. 4a). Through RNA velocity analysis, we found that in the process of germ cell differentiation, some spermatogonia might not differentiate but de-differentiate or stagnate abnormally; at the same time, sperm differentiation was also abnormal (Fig. 4b). In the analysis of cell potential, the differentiation potential of most germ cells decreased with spermatogenesis (higher values corresponded to lower potential) (Fig. 4c). In the analysis of the acceleration of cell differentiation, we found that the acceleration decreases rapidly starting from the late-stage round spermatids (Fig. 4d).

### Differential analysis of Leydig and Sertoli cells revealed metabolic dysfunction in the KO testis

**Fig. 5.**
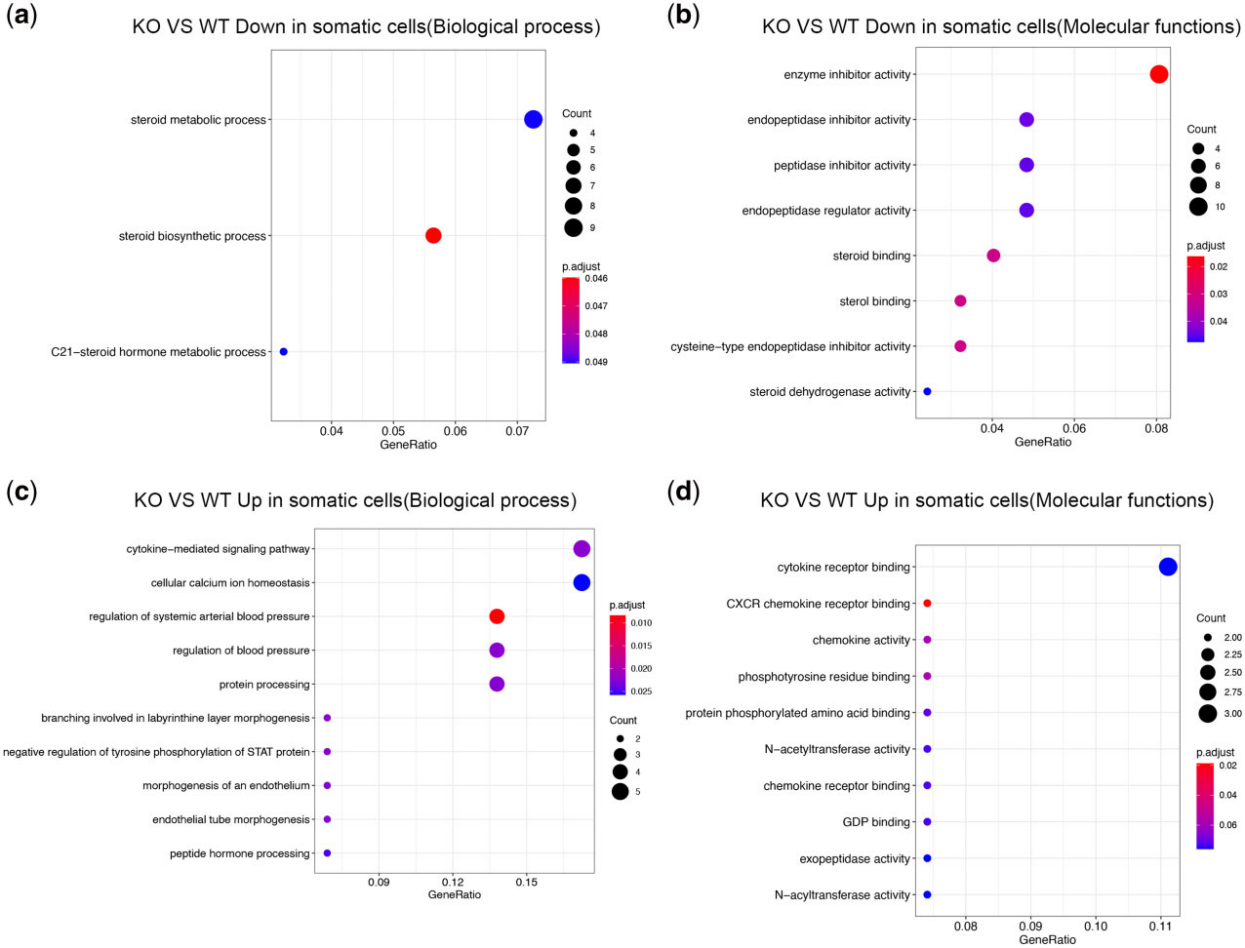
a) Biological process enrichment analysis of genes with down-regulated somatic cells in *Alkbh5* KO mice compared with WT mice. b) GO molecular function enrichment analysis of genes with downregulated somatic cells in *Alkbh5* KO mice compared with WT mice. c) Biological process enrichment analysis of genes with upregulated somatic cells in *Alkbh5* KO mice compared with WT mice. d) GO molecular function enrichment analysis of genes with upregulated somatic cells in *Alkbh5* KO mice compared with WT mice.

Somatic cells (Sertoli cells and Leydig cells) play critical roles in spermatogenesis (Shen *et al.* 2019). We performed differential gene expression and functional enrichment analyses on these 2 cell types. The KO mice had 31 significantly upregulated genes and 141 downregulated genes, in the somatic cells compared to that in the WT nice. The downregulated genes in the KO sample were mainly enriched in steroid secretion-related functions, indicating that steroid secretion decreased with *Alkbh5* KO (Fig. 5, a and b). The upregulated genes in the KO sample were enriched in hormone signaling pathways; this could explain the significant increase in the number and proportion of Leydig cells and Sertoli cells in the KO sample (Fig. 5, c and d).

### Differential analysis of macrophages and lymphoid cells revealed the effects of ALKBH on immunity and anti-inflammation

**Fig. 6.**
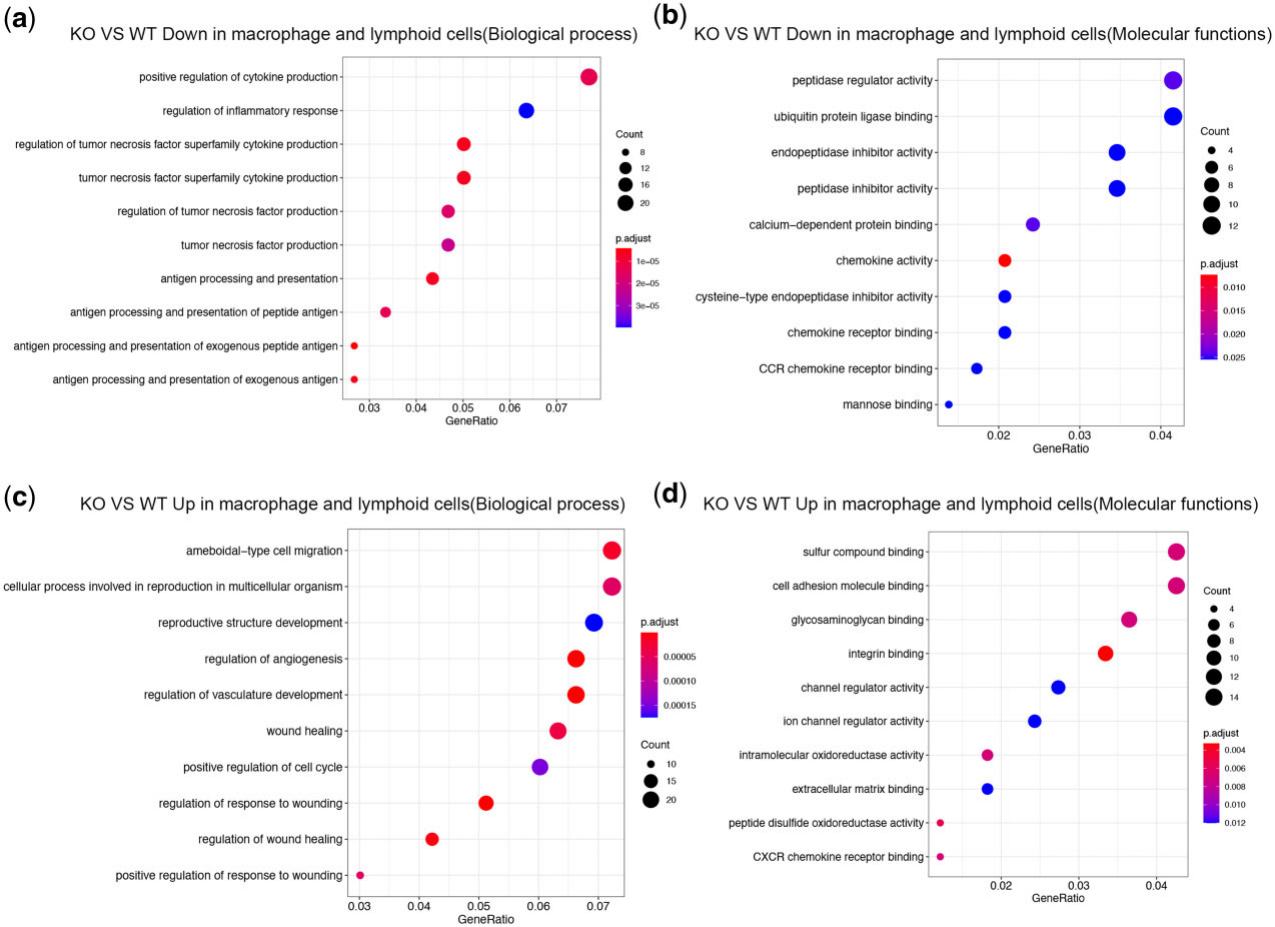
a) Biological process enrichment analysis of genes with downregulated macrophage and lymphoid cells in *Alkbh5* KO mice compared with WT mice. b) GO molecular function enrichment analysis of genes with downregulated macrophage and lymphoid cells in *Alkbh5* KO mice compared with WT mice. c) Biological process enrichment analysis of genes with up-regulated macrophage and lymphoid cells in *Alkbh5* KO mice compared with WT mice. d) GO molecular function enrichment analysis of genes with upregulated macrophage and lymphoid cells in *Alkbh5* KO mice compared with WT mice.

The number and proportion of macrophages and lymphoid cells increased after *Alkbh5* KO. Functional analysis of differentially expressed genes in these cells showed that the KO mice had 355 significantly upregulated genes and 336 downregulated genes, in macrophages and lymphoid cells, when compared to that in the WT mice. The downregulated genes in the KO sample were mainly involved in the regulation of tumor necrosis factor (TNF) and the inflammatory response (Fig. 6, a and b). The functional pathways associated with the upregulated genes in the KO sample were mainly involved in damage repair and tissue regeneration (Fig. 6, c and d).

### CellChat analysis of WT and KO testes revealed different cell interaction patterns

**Fig. 7.**
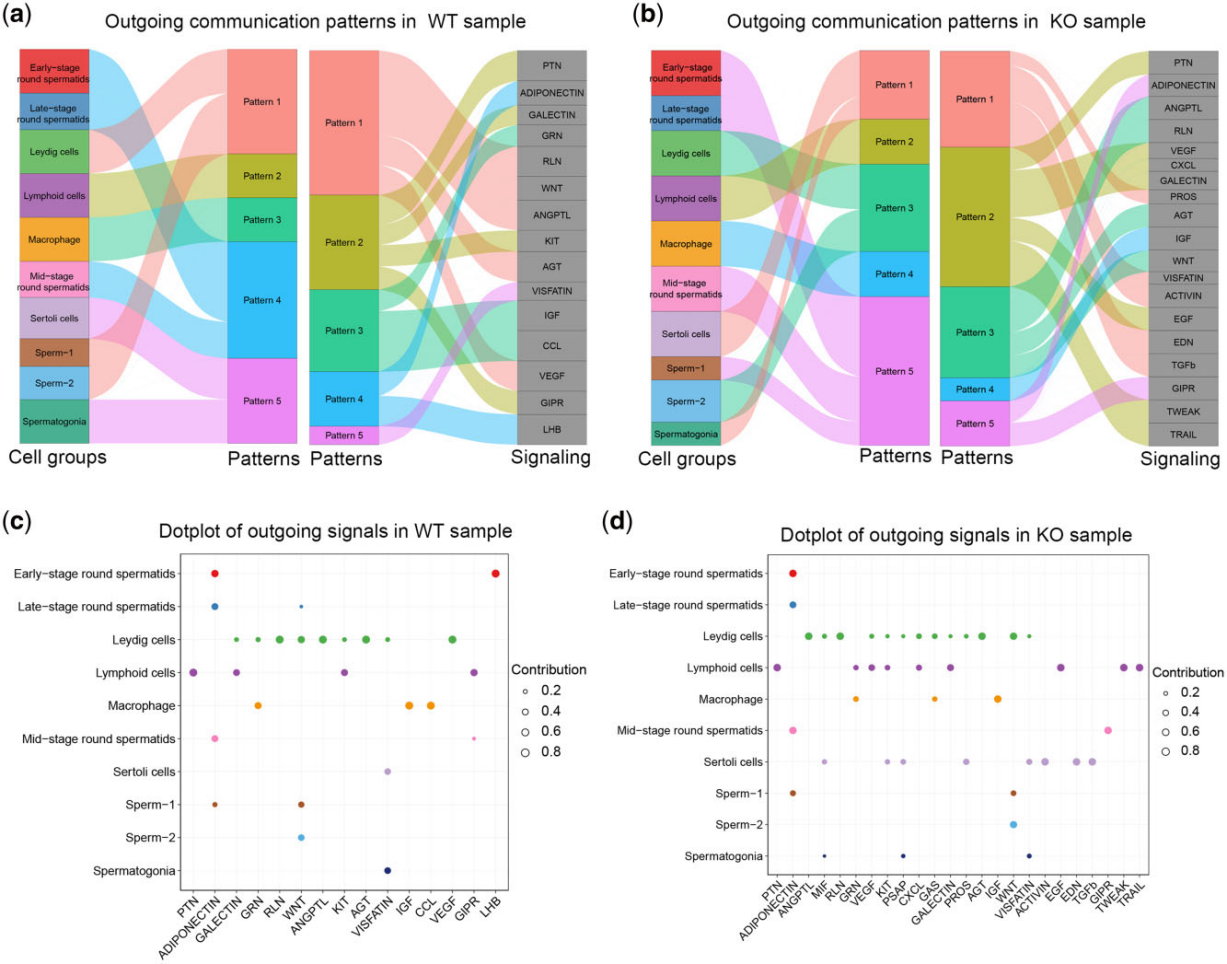
a) Visualization of outgoing communication patterns in WT mice. b) Visualization of outgoing communication patterns in KO mice. c) Dotplot of outgoing signals in different populations in WT mice. d) Dotplot of outgoing signals in different populations in KO mice.

To investigate the interaction between different cell types in WT and KO samples, we performed further analysis by CellChat. The analysis results showed that most of the outgoing signal patterns of WT and KO were similar, but some signals were different. Both WT and KO Leydig cells displayed the characteristics of secreting signals such as WNT, KIT, and RLN (Fig. 7, a and b). However, compared with WT, leydig cells also displayed the characteristics of secreting MIF, PROS, GAS, and other signals. While the early-stage round spermatid of WT can signal LHB, KO was unable to do so (Fig. 7, c and d).

### The expression of genes related to metabolism-related functions and spermatogenesis are altered after Alkbh5 KO

**Fig. 8.**
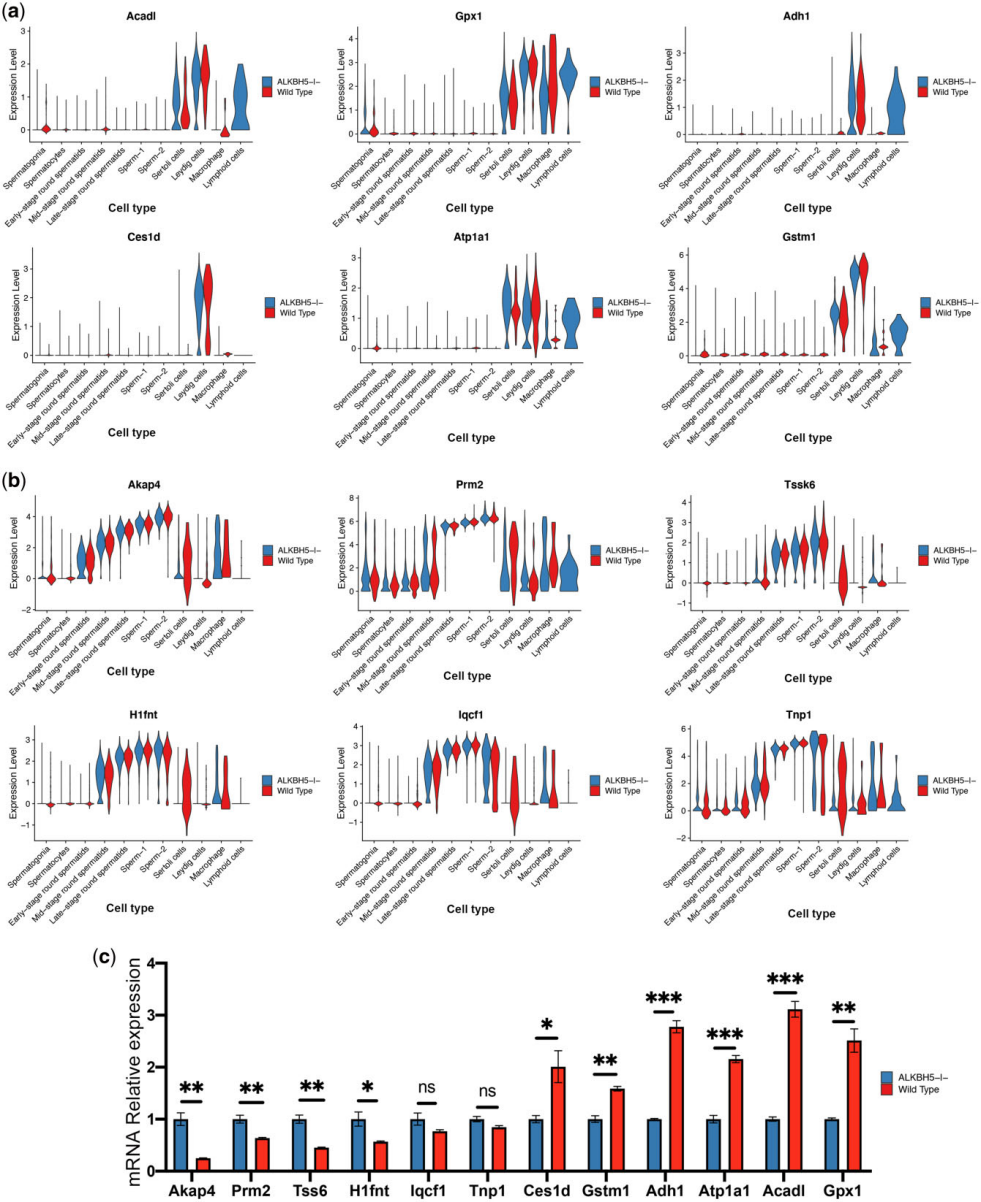
a) Violin plot depicting genes related to metabolism-related functions in different populations. b) Violin plot depicting genes related to spermatogenesis in different populations. c) The mRNA expression of 12 genes in the testicular tissue of *Alkbh5* KO and WT mice was measured by RT-qPCR (***P = 0.0001; **P < 0.01; *P < 0.05; ns, not significant).

Considering the GO analyses results, we decided to further investigate genes related to metabolism-related functions and spermatogenesis. We found that the metabolism-related genes *Acadl*, *Gpx1*, *Adh1*, *Ces1d*, *Atp1a1*, and *Gstm1* were differentially expressed in different cell populations in KO and WT samples, indicating the effect of *Alkbh5* KO on metabolic pathways (Fig. 8a). On the other hand, the genes related to spermatogenesis, *Akap4*, *Prm2*, *Tssk6*, *H1fnt*, *Iqcf1*, and *Tnp1*, were also differentially expressed in different cell populations in KO and WT samples, and the expression of these genes in both WT and KO changed from spermatogonia to sperms in a progressively increasing trend (Fig. 8b). To analyze the differences of these genes as a whole, we decided to verify them by RT-qPCR. We found that *Acadl*, *Gpx1*, *Adh1*, *Ces1d*, *Atp1a1*, and *Gstm1* were greatly downregulated in the KO mice. In contrast, *Akap4*, *Prm2*, *Tssk6*, and *H1fnt* were upregulated in the KO mice (Fig. 8c).

## Discussion

Through bioinformatic analysis, we have presented a novel framework for comparing KO and WT conditions. We observed notable cell-to-cell transcriptional variation, based on single-cell gene expression data. The proportion and number of somatic cells (Leydig cells and Sertoli cells) in KO mice far exceeded that in WT mice. Leydig cells and Sertoli cells were traditionally considered to produce hormones; subsequently, they were found to play a role in the development of germ cells (Yin *et al.* 2017). The proportion of spermatogonia in KO samples was higher than that in the WT samples. However, the number and proportion of cell types in the later stages of germ cell differentiation, such as in sperm, were much lower than that in the WT samples. These findings confirmed that *Alkbh5* KO in mice greatly reduced the number of pachytene spermatocytes and round spermatids in the spermatogonia (Tang *et al.* 2018). This could be attributed to the decrease of spermatogenic cells in KO mice, resulting in a relative increase in the somatic cell proportion. However, the number of somatic cells in KO mice was much higher than that in WT mice. As the steroid hormones are produced by the somatic cells (Shi *et al.* 2018), therefore, we hypothesized that ALKBH5 deficiency affects steroid secretion, due to spermatogenesis stagnation and steroid hormone release disorder. The increase in the steroid hormone secretion in KO mice could be higher than that in WT mice.

We performed differential gene expression analysis on the KO and WT samples. The effects of *Alkbh5* KO on germ cells, somatic cells, macrophages, and lymphoid cells differed considerably. In macrophages and lymphoid cells, the number of significantly upregulated genes was very similar to the number of significantly downregulated genes when *Alkbh5* gene was knocked out. However, following the *Alkbh5* KO in germ cells, the number of significantly upregulated genes was much higher than that of the significantly downregulated genes. In somatic cells, the number of significantly downregulated genes was much larger than that of the significantly upregulated genes. We determined that the genes related to spermatogenesis were markedly downregulated in KO mice. For instance, *Akap4* is the most abundant protein within the sperm fibrous sheath found in all mammals and is an essential component of the flagellum (Huang *et al.* 2005; Pereira *et al.* 2017). It has been demonstrated that *Akap4* can function as a regulator of the cAMP/PKA and PKC/ERK1/2 pathways in human spermatozoa, which are essential for capacitation and acrosome fusion (Rahamim Ben-Navi *et al.* 2016). In addition, Prm2 transcripts are transcribed at step 7 during spermiogenesis, and cytoplasmic ribonucleoproteins are formed for storage before translation (Mali *et al.* 1989). Protamine levels are modestly reduced in mice lacking *Prm2* genes, which leads to reduced sperm compaction, increased DNA damage, and embryo lethality (Cho *et al.* 2003). Furthermore, *Tssk6* is almost exclusively expressed in the testis of mice and humans (Sosnik *et al.* 2009), and its role in the chromatin remodeling of elongating spermatids is essential for male fertility (Spiridonov *et al.* 2005). Therefore, ALKBH5 as a demethylase may have a cell-type-specific effect on the spermatogenesis-related genes expression levels.

Knocking out *Alkbh5* led to abnormal secretion of TNF in the testis, as observed through the analysis of immune cells (macrophages and lymphoid cells). The damage repair pathways were upregulated in these cells. TNF is produced under pathological conditions (Madsen *et al.* 2016). Therefore, these results indicate that knocking out *Alkbh5* caused the testis to fall into a serious pathological state, which stimulated the production of immune cells. In addition, the differentially expressed genes associated with spermatogenesis were functionally enriched in reproduction, regulation of the immune system process, and inflammatory response pathways. Some signals were found to be different in WT and KO samples. LHB has been found to be highly expressed in the testicles of yak, suggesting that LHB might play an integral role in spermatogenesis and steroid synthesis (Kalwar *et al.* 2019). Deleted genes encoding LHB in mice resulted in hypogonadal mice that are infertile, have low levels of testosterone, and experience arrest of spermatogenesis at the round spermatid stage (Lei *et al.* 2001; Zhang *et al.* 2001; Ma *et al.* 2004). In this study, compared to WT mice, KO mice did not have LHB in the early-stage round spermatid. In conclusion, *Alkbh5* influences the stemness of the spermatogonia, the functions of the sperms, and the normal translation process.

The majority of studies on m6A modification mainly focused on posttranscriptional regulation for cytoplasmic mRNA stability, variable shearing, and protein translation (Gheller *et al.* 2020). However, the m6A modification plays an important regulatory role at the gene transcription level (Shi *et al.* 2017): m6A can activate gene transcription by altering the histone modification and chromatin structure (Wang *et al.* 2018). Purification of nuclear RNA in mouse embryonic stem cells using RNA-seq and methylated RNA immunoprecipitation sequencing (MeRIP-seq) demonstrated chromatin changes related to the RNA level of m6A modification. It identified an open chromatin state change, influencing the proliferation and differentiation of embryonic stem cells in mice (Liu *et al.* 2020). m6A modification exists on the mRNA in the cytoplasm and on the chromatin-associated RNA in the nucleus, playing an important regulatory role at the transcriptional level. The upregulated genes in the KO samples were mainly concentrated in mRNA and chromatin-related functional pathways (Hong *et al.* 2022). We speculate that during spermatogenesis, the m6A mRNA modification level and the associated binding proteins of the spermatogenic cells at the different stages regulate the chromatin opening state and the transcription level of downstream genes. This plays an important role in the smooth progression of spermatogenesis. Therefore, abnormal regulation of m6A mRNA modification can lead to male infertility.

In summary, this study revealed the main cell types and maturation processes in the WT mouse testis. In addition, it compared the maturation dysfunction of the testicular cells in the *Alkbh5* KO mouse. This could provide valuable information for further studies on male sterility caused by *Alkbh5* KO. These single-cell transcriptome datasets could play a role in facilitating the diagnosis and discovery of potential causes of male infertility. scRNA-seq enabled analyzing changes in gene expression at the cellular level. However, functional studies that are more detailed are needed to better elucidate the role of ALKBH5 in spermatogenesis.

## Data availability

The raw data have been successfully uploaded and GEO accession number is GSE190396 (https://www.ncbi.nlm.nih.gov/geo/query/acc.cgi?acc=GSE190396).

Supplemental material is available at *G3* online.

## Supplementary Material

jkac130_Supplemental_Figures_and_Table_S1

jkac130_Supplemental_Table_S2
