# Supplementary material for: Comparative analysis of the testes from wild-type and Alkbh5-knockout mice using single-cell RNA sequencing
Source: G3 (Bethesda). 2022 Jun 2;12(8):jkac130. doi: 10.1093/g3journal/jkac130 (PMC9339272; doi:10.1093/g3journal/jkac130)
Supplement: jkac130_Supplemental_Figures_and_Table_S1 [file jkac130_supplemental_figures_and_table_s1.docx]

**Figure S1**

**Figure S2**

**Figure S3**

**Figure S4**

**Figure S1.** The violin plot showing the data quality of WT and Alkbh5 KO mice.

**Figure S2.** Heatmap showed the differentially expressed genes of each sample.

**Figure S3.** Cellular Component enrichment analysis of genes with down-regulated germ cells in Alkbh5 KO mice compared with WT mice(right). GO molecular function enrichment analysis of genes with down-regulated germ cells Alkbh5 KO mice compared with WT mice(left).

**Figure S4.** Cellular Component enrichment analysis of genes with up-regulated germ cells in Alkbh5 KO mice compared with WT mice(right). GO molecular function enrichment analysis of genes with up-regulated germ cells Alkbh5 KO mice compared with WT mice(left).

**Table S1** Sequences of primers used for RT-qPCR analysis.

| Name | Sequences Product size (bp) |
| --- | --- |
| β-Actin: F:5' GGCTGTATTCCCCTCCATCG 3'  Acadl: F:5' TCTTTTCCTCGGAGCATGAC 3'  Gpx1: F:5' AGTCCACCGTGTATGCCTTCT 3'  Ces1d: F:5' ATGCGCCTCTACCCTCTGATA 3'  Adh1: F:5' GCAAAGCTGCGGTGCTATG 3'  Atp1a1: F:5 'GGGGTTGGACGAGACAAGTAT 3'  Gstm1: F:5' ATACTGGGATACTGGAACGTCC 3'  Akap4: F:5' AGAGGGCGAATTAAACCTGGA 3'  Prm2: F:5' ATGGTTCGCTACCGAATGAGG 3'  Tssk6: F:5' CGGGCGACAAACTCCTGAG 3'  H1fnt: F:5' GGCGCAGAACTTACGATCCA 3'  Iqcf1: F:5' ACTTAATGCACCTACTGACGATG 3'  Tnp1: F:5' ACCAGCCGCAAGCTAAAGAC 3' | R:5' CCAGTTGGTAACAATGCCATGT 3' 154  R:5' GACCTCTCTACTCACTTCTCCAG 3' 113  R:5' GAGACGCGACATTCTCAATGA 3' 105  R:5' AGCAAATCTCAAGGAGCCAAG 3' 198  R:5' TCACACAAGTCACCCCTTCTC 3' 225  R:5' CGGCTCAAATCTGTTCCGTAT 3' 173  R:5' AGTCAGGGTTGTAACAGAGCAT 3' 349  R:5' GAGGACACTTATGGGGTCAGA 3' 139  R:5' CTCCGCCTTCTGCATGACC 3' 221  R:5' ACCGTCCCTTTATACTTCTTGGA 3' 109  R:5' GACTTCCCCTCGTGGTGAG 3' 215  R:5' CCGACGTACCAGTGTACCG 3' 160  R:5' TTTCCTACTTTTCAGGACGCTC 3' 120 |
